# Root dentine thickness of danger zone in mesial roots of mandibular first molars

**DOI:** 10.1186/s12903-020-1026-8

**Published:** 2020-02-06

**Authors:** Guangchao Zhou, Diya Leng, Mingming Li, Yang Zhou, Cuifeng Zhang, Chao Sun, Daming Wu

**Affiliations:** 1 Jiangsu Key Laboratory of Oral Diseases, Nanjing Medical University, 136 Hanzhong Road, Nanjing, 210029 People’s Republic of China; 2 Department of Radiology, the Affiliated Stomatological Hospital of Nanjing Medical University, 136 Hanzhong Road, Nanjing, 210029 People’s Republic of China; 3 Department of Endodontics, the Affiliated Stomatological Hospital of Nanjing Medical University, 136 Hanzhong Road, Nanjing, 210029 People’s Republic of China

**Keywords:** Cone-beam computed tomography, Danger zone, Mandibular first molars, Mesial roots

## Abstract

**Background:**

Better understanding of the danger zone anatomy in mesial roots (MRs) of mandibular first molars (MFMs) may serve to decrease the risk of mishaps. This study aimed to measure the minimal distal dentine thicknesses of danger zone in MRs of MFMs in a native Chinese population using cone-beam computed tomography (CBCT).

**Methods:**

CBCT images of 1792 MFMs from 898 Chinese patients were analyzed. The minimal distal dentine thicknesses of the mesiobuccal (MB) and mesiolingual (ML) canals below the furcation 1, 2, 3, 4, 5 mm were measured. The association between the minimal distal dentine thicknesses and the root lengths, patient’s age and gender, side were assessed.

**Results:**

The minimal distal dentine thicknesses of MB and ML canals are located 3 ∼ 4 mm below the furcation for both men and women. There are no differences between MB and ML canals, while the minimal distal dentine thicknesses of MB and ML canals were higher in men than women (*P* < 0.05), except at 1 and 3 mm of ML canals (*P* > 0.05). The minimal distal dentine thicknesses of MB and ML canals increased with age in both men and women at each location (*P* < 0.05). The minimum distal dentine thickness at every location were significantly different between long teeth and short teeth both in men and women (*P* < 0.05), with short teeth having the smallest mean values. There are no significant differences between two sides (*P* > 0.05).

**Conclusions:**

The minimal distal dentine thicknesses of MRs in MSMs have close correlation with root length, patient’s age and gender.

## Background

The mandibular first molars (MFMs) are the first posterior teeth erupt, they are more likely to be affected by lesion. MFMs seem to be the most frequent endodontically treated teeth, with an incidence as high as 17.0% [1]. They usually have 2 or 3 roots, with 2 or 3 canals in the mesial roots (MRs) [2]. Approximately 2 mm below the furcation of MFMs, the MRs have a greater concavity in distal surface and the thickness of dentine is limited [3]. They are described as danger zones because there are more prone to strip perforation during root canal shaping and post space preparation procedures [4, 5]. In addition, the excessive structure loss in danger zones may also lead to root fracture under functional loads. These complications make the root canal system connecting with its support tissue, promoting the spread of bacteria and inflammatory reactions that can hinder the success of endodontic treatment [6]. Therefore, the knowledge of the root and canal morphology and dentin thickness in the danger zones of MFMs is essential for preventing endodontic mishaps leading to failure [7].

Many studies have investigated the danger zone of the MRs of MFMs in different human races, such as Italy [3], Spain [8], Brazil [5, 9], USA [10–12] and Asian origin [7, 13, 14]. In general, the danger zone is located 4 to 6 mm below the canal chamber orifice [10], and the minimum distal dentin thickness was located between 1 and 2 mm under the furcation. The mean thickness of dentine 2 mm below the furcation in MRs of mandibular molars ranges from 0.78 to 1.27 mm [5, 7–14]. In addition, there are some reports correlating these measurements with root length of the teeth. Sauáia et al. [5] reported that there was a significant difference in the minimum thickness of the distal root wall of the mesiobuccal (MB) canal of MFMs 2 mm below the furcation between long root teeth and short root teeth. The thinnest walls and the deepest concavities in the distal walls of the MRs were found in the longest teeth. Therefore, they suggest that long molars may have a higher risk of strip perforation in MB canals if flared to a larger size [5]. Dwivedi et al. [14] also reported that the distal wall thickness and distal concavity of the MRs of the MFMs were found to be thinner in longer teeth compared with shorter teeth, with the difference approximate 0.8 mm. However, although the thickness of the dentine in the danger zone has been studied widely, there are little information in the literatures correlating these measurements with other features of the teeth, such as patient’s age and gender.

Many kinds of methods have been used to assess the minimum dentine thickness of danger zone, such as radiographs, serial sectioning [5, 7], Micro-computed tomographic (Micro-CT) [12]. Radiographs were not a reliable method for measuring residual thickness of tooth walls, because they showed greater thicknesses than were actually present [15]. Serial sectioning is destructive, so it can’t be used in vivo and the samples can’t be used for further studies. Micro-CT provide detailed information about the dentine thicknesses, canal morphology and curvatures in micrometer intervals [12]. It with novel software also provide valuable anatomical information for optimizing instrumentation and minimizing mishaps in nonsurgical root canal treatment [16]. However, Micro-CT has high radiation doses and limited specimen size, it could not be used to scan the head of a living human, which limits its clinic application. Cone-beam computed tomographic (CBCT) imaging provides high-quality, accurate, nondestructive 3-dimensional system for proper information and identification of internal root canal anatomy [17]. It can be a powerful tool in endodontic diagnosis, treatment planning and follow-up [18]. CBCT imaging could measure dentine thickness accurately [19]. Therefore, the aim of this study was to assess the minimum distal dentine thickness of danger zone using CBCT and to analyze the correlation between the dentine thickness and root length, age and gender, side of the MFMs in Chinese population.

## Methods

### CBCT images collections

The Ethical Committee Department of the Affiliated Stomatological Hospital of Nanjing Medical University approval was obtained (PJ2017–053-001). Written informed consents were acquired from all patients. CBCT images scanned for endodontic, orthodontic or implant treatment, diagnosis of impacted teeth and facial trauma were randomly collected from the Department of Radiology, the Affiliated Stomatological Hospital of Nanjing Medical University from Aug 2017 to Dec 2018. The CBCT images showed the MFMs clearly. The samples were selected according to the following exclusion criteria: (1) MFMs with unformed apices, root resorption or fractures. (2) MFMs with complicated root canal morphology or calcification that root canals cannot be identified clearly. (3) The presence of caries, periapical or periradicular lesions, or any other odontogenic or nonodontogenic pathology. (4) MFMs treated by root canal filling, posts or crowns restoration. (5) Artifacts from adjacent implants or metal crowns which made the measurement can’t be carried out.

CBCT images of 1792 MFMs from 898 individuals (445 men and 453 women) were selected. The age of the patients ranged from 18 to 89 years, with mean age of 43.39 ± 13.96 years for men and 40.14 ± 14.00 years for women. The patients were stratified into 3 categories: 18–30 years group, 31–50 years group and ≥ 51 years group.

### CBCT images evaluations

MFMs were imaged with a CBCT scanner (NewTom VG, QR srl., Verona, Italy) at 110 kVp and 3.6 ∼ 4.8 mA with a voxel size of 0.2 mm and field of view of 12 × 8 cm or 15 × 15 cm by an experienced radiologist according to the manufacturer’s recommended protocol.

Images were assessed by two experienced endodontists using NNT 4.6 software (QR srl., Verona, Italy), which can adjust the contrast and brightness to achieve optimal visualization. Before the experimental reading, their measurements were calibrated by reviewing 20 CBCT images of MFMs selected to ensure the veracity of the values. The intra and inter-examiner reliability were assessed by Cohen’s kappa statistical analysis. The kappa values for the intra-examiner and inter-examiner agreements were 0.877 to 0.933, respectively.

**Fig. 1:**
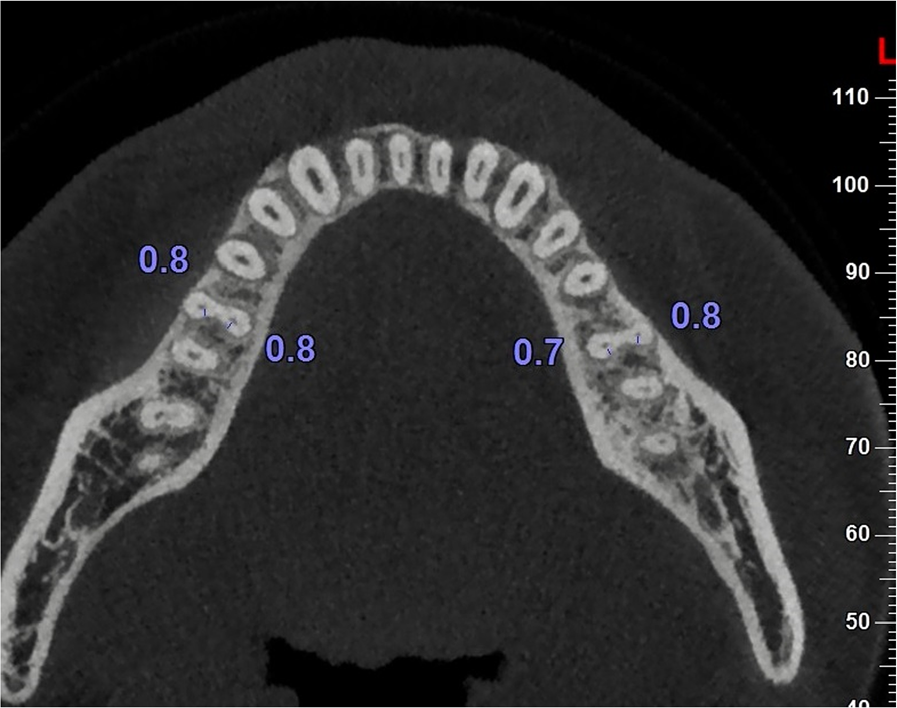
Measurement of the minimum distal dentine thickness of danger zone on CBCT images

The MR length of each specimen was recorded from the furcation of MFMs to the apex using CBCT axial planes, and categorized according to the length of teeth as follows: long root (> 10.0 mm), medium root (9.0 ∼ 10 mm), short root (< 9.0 mm). The minimum distal wall thickness of the MB and mesiolingual (ML) canals were measured according to previous studies [20]. Briefly, 1, 2, 3, 4, 5 mm under the furcation, the minimum distal wall thickness of the MB and ML canals of the MRs was measured in axial planes by measuring the minimum distance from the edge of the root canal to the external surface of the root distal concavity (Fig. 1). All measurements were performed at 4 magnifications using the NNT software. The thickness was measured three times, and the mean thickness was recorded.

### Statistical analysis

The association between the minimum distal wall thickness and the root length, patient’s age and gender, side were assessed using the Wilcoxon and the Kruskal-Wallis test with the SPSS 22.0 (IBM Corp., Armonk, NY, USA). *P* < 0.05 was considered statistically significant.

## Results

### Association between the minimum distal dentine thickness and the patient’s age and gender

The association between the minimum distal dentine thickness of MB and ML canals and the patient’s age and gender are showed in Tables 1 and 2.

**Table 1:** The minimal distal dentine thicknesses of MB canals in danger zone (mean ± SD, mm)

Groups	Men					Women				
	1 mm	2 mm	3 mm	4 mm	5 mm	1 mm	2 mm	3 mm	4 mm	5 mm
18–30 y	0.95 ± 0.15	0.80 ± 0.14	0.78 ± 0.13	0.80 ± 0.14	0.78 ± 0.14	0.89 ± 0.14	0.76 ± 0.14	0.74 ± 0.13	0.76 ± 0.14	0.76 ± 0.15
31–50 y	0.96 ± 0.17	0.82 ± 0.15	0.80 ± 0.14	0.79 ± 0.14	0.81 ± 0.13	0.93 ± 0.16	0.79 ± 0.14	0.79 ± 0.13	0.80 ± 0.14	0.80 ± 0.14
≥51 y	1.01 ± 0.18	0.85 ± 0.15	0.82 ± 0.15	0.83 ± 0.15	0.83 ± 0.15	0.97 ± 0.16	0.84 ± 0.14	0.81 ± 0.14	0.81 ± 0.15	0.82 ± 0.14
Total	0.98 ± 0.17	0.83 ± 0.15	0.80 ± 0.14	0.81 ± 0.14	0.81 ± 0.14	0.93 ± 0.16	0.79 ± 0.14	0.78 ± 0.14	0.79 ± 0.14	0.79 ± 0.13

**Table 2:** The minimal distal dentine thicknesses of ML canals in danger zone (mean ± SD, mm)

Groups	Men					Women				
	1 mm	2 mm	3 mm	4 mm	5 mm	1 mm	2 mm	3 mm	4 mm	5 mm
18–30 y	0.98 ± 0.14	0.82 ± 0.13	0.79 ± 0.12	0.79 ± 0.13	0.80 ± 0.10	0.98 ± 0.16	0.80 ± 0.12	0.77 ± 0.12	0.77 ± 0.13	0.78 ± 0.13
31–50 y	1.00 ± 0.17	0.84 ± 0.15	0.79 ± 0.13	0.79 ± 0.12	0.80 ± 0.13	0.99 ± 0.17	0.82 ± 0.14	0.80 ± 0.13	0.79 ± 0.12	0.80 ± 0.13
51–60 y	1.02 ± 0.17	0.86 ± 0.15	0.82 ± 0.14	0.82 ± 0.13	0.83 ± 0.12	1.02 ± 0.17	0.84 ± 0.14	0.81 ± 0.13	0.82 ± 0.14	0.82 ± 0.13
Total	1.01 ± 0.17	0.84 ± 0.15	0.80 ± 0.13	0.80 ± 0.13	0.81 ± 0.12	0.99 ± 0.17	0.82 ± 0.13	0.79 ± 0.13	0.79 ± 0.13	0.80 ± 0.13

The minimal distal dentine thicknesses of MB and ML canals are located 3 ∼ 4 mm below the furcation for both men and women. There are no differences between MB and ML canals, while the minimal distal dentine thicknesses of MB and ML canals were higher in men than women (*P* < 0.05), except at 1 and 3 mm of ML canals (*P* > 0.05).

The minimal distal dentine thicknesses of MB and ML canals increased with age in every age group in both men and women at each location (*P* < 0.05).

### Association between the minimum distal dentin thickness and the root lengths

The association between the minimum distal dentine thickness and the root lengths are showed in Table 3. The minimum distal dentine thickness at every location was significantly different between long teeth and short teeth both in men and women (*P* < 0.05), with short teeth being smallest.

**Table 3:** The minimal distal dentine thicknesses of mesial roots in danger zone according to root lengths (mean ± SD, mm)

Groups	Men					Women				
	1 mm	2 mm	3 mm	4 mm	5 mm	1 mm	2 mm	3 mm	4 mm	5 mm
long	0.97 ± 0.17	0.82 ± 0.13	0.78 ± 0.12	0.78 ± 0.11	0.78 ± 0.11	0.97 ± 0.16	0.82 ± 0.13	0.79 ± 0.14	0.82 ± 0.14	0.82 ± 0.12
medium	0.93 ± 0.15	0.79 ± 0.13	0.76 ± 0.12	0.76 ± 0.12	0.78 ± 0.11	0.91 ± 0.14	0.77 ± 0.12	0.75 ± 0.11	0.75 ± 0.12	0.76 ± 0.12
short	0.92 ± 0.14	0.76 ± 0.13	0.74 ± 0.11	0.75 ± 0.12	0.75 ± 0.11	0.87 ± 0.13	0.73 ± 0.13	0.71 ± 0.11	0.73 ± 0.11	0.74 ± 0.12

### Association between the minimum distal dentine thickness and side

At every location, there are no significant differences between right and left side MFMs (*P* > 0.05) (Table 4).

**Table 4:** The minimal distal dentine thicknesses of mesial roots in danger zone according to side (mean ± SD, mm)

Groups	Men					Women				
	1 mm	2 mm	3 mm	4 mm	5 mm	1 mm	2 mm	3 mm	4 mm	5 mm
Left	0.94 ± 0.15	0.78 ± 0.13	0.76 ± 0.12	0.76 ± 0.12	0.77 ± 0.11	0.90 ± 0.15	0.76 ± 0.13	0.74 ± 0.13	0.75 ± 0.12	0.76 ± 0.12
Right	0.94 ± 0.15	0.80 ± 0.13	0.76 ± 0.11	0.76 ± 0.12	0.78 ± 0.11	0.91 ± 0.14	0.76 ± 0.12	0.74 ± 0.11	0.76 ± 0.12	0.76 ± 0.12

## Discussion

Root and canal morphology of permanent teeth showed close associations with age and gender [21, 22]. The pulp-dentinal complex change over the course of a lifetime with physiological deposition of secondary dentine, which contributing to a reduction of the pulp chamber size and root canal diameter [23, 24]. Consequently, canals were sharply defined and narrow, sometimes too narrow in older adults, while young patients tend to have large single canals and pulp chambers [23]. In addition, the cementum deposition with time in people and peaks in old age, resulting in a complex and changeful root morphology in old age [25]. Therefore, it is accepted that calcific changes of the pulp-dentinal complex over time pose challenges for the clinician [26].

The dangerous zone of the root canal preparation is the weakest thickness zone of the root canal wall. Root thickness tends to decrease considerably in danger zone during root canal shaping. It is particularly prone to excessive weakness and undesirable side effects [10], especially Nickel-titanium (NiTi) instruments are extensively used in endodontic treatment [27]. Stress concentration of tooth root should be concerned during the dental treatment, because it is closely related to vertical root fracture. In term of stress concentration, canal curvature seems more important than external root morphology, and that reduced dentine thickness increases the magnitude but not the direction of maximum tensile stress [28]. Versluis et al. [29] reported that external distal and mesial surfaces of roots with oval canals showed moderate stress concentrations that were minimally affected by preparations, while stress concentrations emerged on roots with round canals when preparation sizes increased. Therefore, better understanding of the danger zone anatomy may serve to decrease the risk of mishaps.

There are some reports on the radicular wall thicknesses of danger zone in MFMs, which showed that the mean thickness of dentin ranges from 0.78 to 1.27 mm, with the minimal thicknesses of 0.4 mm [3, 7, 9–11, 13, 30]. For example, Bryant et al. [31] reported that the mean size of the danger zone for 200 canals used was 0.79 mm. Keles et al. [32] reported that the thinnest canal walls of MB canals were 1.16 ± 0.20 mm and ML canals were 1.19 ± 0.18 mm. De-Deus et al. [33] found that the danger zone values in the MB canals varied from 0.67 to 1.93 mm with an average of 1.13 ± 0.21 mm, and in the ML canals varied from 0.77 to 1.89 mm with an average of 1.10 ± 0.21 mm, locating up to 4 mm under the furcation area. These results vary slightly because the researchers had used different methods of measuring the thickness of the root canal wall in the danger zone, and they selected different ranges of the danger zone or different human species for studies. Moreover, there is little information in the literatures correlating these measurements with other features of the teeth, such as patient’s age and gender.

In the present study, the minimal distal dentine thicknesses associated with the MB and ML canals below the furcation 1, 2, 3, 4, 5 mm of Chinese population were measured. The results showed that the minimal distal dentine thicknesses of MB and ML canals are located 3 ∼ 4 mm below the furcation for both men and women, with a mean range of 0.78 ∼ 0.80 mm, and there are no differences between MB and ML canals. The result indicated that the danger zone of MFMs is located at the same position for both men and women.

In the present study, the minimal distal dentine thicknesses of MB and ML canals were higher in men than women (*P* < 0.05), except at 1 and 3 mm of ML canals (*P* > 0.05). These results confirm that the minimal distal dentine thicknesses of MB and ML canals with differs between men and women. Gender is an important factor to influence the distal wall thickness of the MRs of the MFMs. MFMs of women are more probability to strip perforation during root canal shaping and post space preparation procedures. Therefore, thinner or smaller instruments are suitable for women during endodontic treatment and post space preparation procedures.

The results of this study showed that the minimal distal dentine thicknesses of MB and ML canals increased with age in every age group in both men and women at each location (*P* < 0.05). Age is another important factor to influence the distal wall thickness of the MRs of the MFMs. MFMs of younger people have larger canals and thinner root canal walls than these of older people.

The results of this study showed that the minimum distal dentine thickness at every location was significantly different between long teeth and short teeth both in men and women (*P* < 0.05), with short teeth being smallest. These results are different with previous reports by Sauáia et al. [5] and Dwivedi et al. [14] in which the distal wall thickness and distal concavity of the MRs of the MFMs were found to be thinner in longer teeth compared with shorter teeth. Possible explanation is that ethnic difference is an important factor to influence the distal wall thickness of the MR of the MFMs.

The decrease of the dentine thickness is an important point during the evaluation of root canal instrumentation because excessive enlargement of the root canal space can lead to accidents such as perforations. According to Lim and Stock, 200~300 μm dentine thickness should be retained after preparation in order to withstand compaction forces during obturation and to prevent perforation or vertical root fracture [13]. Based on the results of the present study, root canal preparation in danger zone decrease dentine should not more than 0.5 mm, otherwise the possibility of perforation increases. To prevent strip perforations, firstly, the selection of great taper NiTi instruments should be cautious for the “danger zone” of insufficient dentin thickness of root canal wall. Secondly, coronal flaring should be limited and instruments should be directed towards the lateral and mesial canal walls that have much thicker dentine and away from the danger zone [9]. Finally, dentists should pay more attention to shorter teeth of young women during endodontic treatment and post space preparation procedures.

This study provided a detailed description of the distal wall thickness of the MRs of the MFMs in a large sample of a Chinese population. These findings are very important for clinicians because they will help to increase the success rates for endodontic treatment and post space preparation of patients of different gender and ages. In the study, some middle mesial canals (MMCs) of MRs were found in MFMs. However, due to the small number of MMCs in every age group, no measurements were made. In addition, the danger zone was mainly towards the distal region of the roots and towards the mesial region in few MB and ML canals, so the mesial wall thickness was not measured. Further studies will be conducted to investigate these issues.

## Conclusions

Under the limitations of the present study, it may be concluded that the minimal distal dentine thicknesses of MRs in MFMs were higher in men than women, and increased as age advances both in men and women. In addition, the minimal distal dentine thicknesses of MRs in shorter MFMs is thinner than in longer teeth. These results suggested that the use of large taper instruments should be careful to prevent root canal perforation and other complications in patients who are younger or have shorter root lengths, especially in female patients.

## Data Availability

The datasets used and/or analysed during the current study are available from the corresponding author on reasonable request.

## Abbreviations

CBCT: Cone-beam computed tomography
MB: Mesiobuccal
MFMs: Mandibular first molars
Micro-CT: Micro-computed tomographic
ML: Mesiolingual
MMCs: Middle mesial canals
MRs: Mesial roots
NiTi: Nickel-titanium
